# PTCY-Based Haploidentical Donor Transplantation versus HLA-Matched Related and Unrelated Donor Transplantations in Patients with Refractory or Relapsed Lymphoma—A Matched-Pair Analysis

**DOI:** 10.3390/cancers15215246

**Published:** 2023-10-31

**Authors:** Sarah Haebe, Alessia Fraccaroli, Elena Stauffer, Dusan Prevalsek, Anna K. Zoellner, Heidrun Drolle, Hans-Joachim Stemmler, Martin Dreyling, Michael von Bergwelt-Baildon, Johanna Tischer

**Affiliations:** Department of Medicine III, Ludwig-Maximilians-University (LMU) University Hospital Munich, 81377 Munich, Germanymichael.bergwelt@med.uni-muenchen.de (M.v.B.-B.)

**Keywords:** haploidentical stem cell transplantation, high-risk non-Hodgkin lymphoma, matched-pair analysis

## Abstract

**Simple Summary:**

Haploidentical donor hematopoietic stem cell transplantation (Haplo-HSCT) has the potential to cure patients with refractory and relapsed (r/r) B and T cell non-Hodgkin lymphoma (NHL). In this matched-pair study, we compared intermediate and long-term outcomes between Haplo-HSCT and HLA-matched related donor (MRD) and unrelated donor (URD) transplantations in patients with r/r lymphoma using age, disease status, lymphoma classification and performance status as matching criteria. While we found comparable outcome and relapse rates among the three groups after >10 years of median follow-up, patients undergoing Haplo-HSCT exhibited lower acute and chronic graft-versus-host disease incidences. Given the rapid and nearly universal donor availability, we propose that Haplo-HSCT is an effective alternative to URD-HSCT in patients with non-remission disease.

**Abstract:**

Allogeneic hematopoietic stem cell transplantation (allo-HSCT) has demonstrated its potential as a curative option for patients with r/r lymphoma. With the introduction of post-transplant cyclophosphamide-based (PTCY) graft-versus-host disease (GvHD) prophylaxis, allo-HCT using haploidentical related donors (Haplo-HSCT) has emerged as a valuable alternative for patients without an available HLA-matched donor. In this study, we compared intermediate and long-term outcomes between Haplo-HSCT and HLA-matched related donor (MRD) and unrelated donor (URD) transplantations in 16 matched pairs using age, disease status, lymphoma classification and performance status as matching criteria. Of note, 88% of patients in each group presented with active disease at the time of conditioning. After a median follow-up of >10 years, 10-year overall and progression-free survival and non-relapse mortality incidence after Haplo-HSCT were 31%, 25% and 38%, respectively, and did not differ compared to the values observed in MRD-HSCT and URD-HSCT. A remarkable lower incidence of acute GvHD ≥ II and moderate and severe chronic GvHD was observed after Haplo-HSCT compared to MRD-HSCT (50%/50%, *p =* 0.03/0.03) and URD-HSCT (44%/38%, *p =* 0.04/0.08), resulting in slightly higher 10-year GvHD-free and relapse-free survival (25%) and chronic GvHD-free and relapse-free survival (25%) in the Haplo-HSCT group. In conclusion, Haplo-HSCT is an effective treatment in patients with non-remission NHL. Given its advantage of immediate availability, haploidentical donors should be preferably used in patients with progressive disease lacking an HLA-matched related donor.

## 1. Introduction

Novel immunotherapeutic drugs, in particular chimeric antigen receptor (CAR) T cells, have emerged as highly promising treatment options for patients with refractory and relapsed (r/r) non-Hodgkin lymphoma (NHL) [1,2,3], but only a subset of patients show durable responses. In the past, allogeneic hematopoietic stem cell transplantation (allo-HSCT) has demonstrated its potential as a curative option for patients with r/r lymphoma [4,5,6,7,8,9,10]. However, the application of HLA-matched allo-HSCT is still limited by donor availability, especially in ethical minorities, and is not feasible for patients suffering from rapidly progressive disease [11,12,13]. Through the introduction of post-transplantation cyclophosphamide (PTCY) for graft-versus-host disease (GvHD) prophylaxis, T cell replete (TCR) HSCT using a related, haploidentical donor (Haplo) has become a feasible treatment option that has led to a substantial increase in HSCT from haploidentical related donors [14]. Originally, Haplo-HSCT was performed following nonmyeloablative conditioning, but given the high relapse rates more intensive protocols have been developed [15,16]. In the last few years, the concept of reduced-intensity conditioning (RIC) T cell replete (TCR) Haplo-HSCT using PTCY as GvHD prophylaxis has become a well-tolerated and time-saving treatment option for almost all transplant-eligible patients with hematologic malignancies [17,18].

Furthermore, retrospective studies have demonstrated similar outcomes, with lower incidence of chronic GvHD in patients with r/r lymphoma grafted from haploidentical donors (Haplo) compared to patients grafted from HLA-related donors (MRD) or unrelated donors (URD) [19,20,21,22,23,24]. However, disease progression and relapse after Haplo-HSCT is also still a major concern, particularly in patients with non-remission disease prior to HSCT initiation, and long-term outcome data comparing different transplantation settings are still limited. In this study, we wanted to further elucidate the role of Haplo-HSCT in patients with high-risk r/r B and T cell NHL. Thus, we performed a matched-pair analysis using age, disease status, lymphoma classification and performance status as matching criteria to compare long-term outcomes, GvHD incidences and treatment-related toxicities and infections between MRD-HSCT, URD-HSCT and Haplo-HSCT.

## 2. Materials and Methods

### 2.1. Study Design

Eligible were patients (≥18 years) with r/r NHL undergoing non-myeloablative (NMC) or RIC allo-HSCT between 2000 and 2017 at our institution. Patients with r/r B-NHL who did not receive an anti-CD20 antibody prior to allo-HSCT initiation and/or all patients who underwent a previous allo-HSCT were excluded. Eligible donors included MRD, 8/8 or 10/10 HLA-matched URD (allele-level match at HLA-A, -B, -C and -DRB1, -DQB1) or Haplo (related and mismatched ≥ 2 HLA-loci). All patients receiving Haplo-HSCT either lacked HLA-matched donors or required a donor search that was not performable within a reasonable timeframe with respect to the aggressive disease course. Donor-specific antibody (DSA) screening and cytotoxic crossmatch were uniformly performed when patients were evaluated for Haplo-HSCT, whereby Haplo-HSCT donors were considered ineligible if DSAs were found.

Recipients of Haplo-HSCT were limited to those undergoing our center-specific sequential cytoreductive chemotherapy prior to RIC Haplo-HSCT consisting of clofarabine (30 mg/m^2^) for five days, as previously described [8,25,26]. Haplo-HSCT post-grafting immunosuppression consisted of PTCY, tacrolimus (Tac) and mycophenolate mofetil (MMF) [17]. GvHD prophylaxis in MRD-HSCT was limited to calcineurin inhibitor (CNI)-based approaches, while patients undergoing URD-HSCT additionally received anti-thymocyte globulin (ATG) (10–20 mg/kg BW IV daily over 3 days). In the absence of GvHD and toxicity, prophylactic donor lymphocyte infusion (pDLI) was considered after discontinuation of immunosuppression with a starting dose of 1 × 10^5^ CD3+ cells/kg for Haplo-HSCT, 5 × 10^5^ CD3+ cells/kg for URD-HSCT and 1 × 10^6^ CD3+ cells/kg for MRD-HSCT and subsequent escalations.

Pre-defined variables including patient age at HSCT (±5 years), disease status prior to HSCT initiation, lymphoma classification and patient performance status (ECOG and Karnofsky performance scale) served as matching criteria between the three groups (Haplo, MRD, and URD).

### 2.2. Definition

r/r NHL was defined as disease relapse/progression after ≥1 cycle of chemotherapy following a previous autologous HSCT or unresponsive to ≥2 cycles of chemotherapy. Active disease was defined when less than a CR was achieved by the most recent treatment prior to HSCT initiation. The Disease Risk Index (DRI) was defined as reported previously [27]. Consensus criteria were used to define the intensity of the conditioning regimens [28]. Engraftment was defined following the Center for International Blood and Marrow Transplant Research criteria [29]. Staging of acute and chronic GvHD was performed according to consensus standards [30,31]. Non-hematological toxicity was graded according to the recorded National Cancer Institute Common Terminology Criteria for Adverse Events (CTCAE v4.0) from the initiation of the therapy until day + 30 after transplantation. CMV IgG sero-positivity of either the donor or the recipient, or both, was defined as high-risk constellation for CMV reactivation.

### 2.3. Supportive Care

Antimicrobial prophylaxis and infection surveillance were applied according to local center strategies [32]. Prophylaxis of veno-occlusive disease was performed with continuous infusion of low-dose unfractionated heparin and orally applied ursodeoxycholic acid. Hyperhydration, sodium bicarbonate and Mesna were applied with cyclophosphamide for prophylaxis of hemorrhagic cystitis.

### 2.4. Statistical Methods

The Haplo-HSCT group was compared against (1) the MRD-HSCT and (2) URD-HSCT groups. Overall survival (OS) was defined as the time from allo-HSCT without death from any cause. Progression-free survival (PFS) was defined as the time from transplantation without progression of disease/relapse or death. Cumulative incidences (CIs) for relapse/progression and non-relapse mortality (NRM) were calculated considering both events as competing risks. Death and relapse/progression were considered as competing events for acute and chronic GvHD. GvHD-free and relapse-free survival (GRFS) was defined as the time from transplantation without the development of grade III–IV acute GvHD or chronic GvHD requiring systemic treatment, relapse or death, and chronic GvHD-free and relapse-free survival (CRFS) was defined as the time from transplantation without the development of moderate or severe chronic GvHD (according to National Institutes of Health consensus criteria), relapse or death, as previously described [33]. Probabilities of OS, PFS, GRFS and CRFS were calculated using the Kaplan–Meier estimates. Patient-, disease- and transplant-related features were compared among the three groups using the Chi-square test for categorical variables and the Kruskal–Wallis test for continuous variables. All statistical analysis was performed using GraphPad version 8 (Boston, MA, USA) and R software version R.3.4.4.

## 3. Results

### 3.1. Patients and Transplant Characteristics

Among thirty-six recipients of Haplo-HSCT, sixteen patients could be successfully pair-matched with sixteen MRD-HSCT and sixteen URD-HSCT recipients using patient age at HSCT (±5 years), disease status prior to HSCT initiation, lymphoma classification and patient performance status (ECOG and Karnofsky performance scale) as matching criteria between the three groups (Haplo, MRD, and URD).

All patients were heavily pretreated, and most patients presented with a high to very high modified DRI score and a high HCT-CI score. Moreover, 88% of patients in each group presented with active disease at the start of conditioning. Patient characteristics are summarized in Table 1.

Haplo donors were children (n = 10), siblings (n = 4), parents (n = 1) and other relatives (n = 1) and the median donor age was 27 y (range: 19–68 y). Bone marrow (BM) was the preferred graft source in the Haplo-HSCT group, according to the original Baltimore protocol, while most of the MRD/URD transplants were performed with peripheral blood stem cell grafts (*p <* 0.001). There was no significant difference in terms of lymphoma histology and ABO incompatibility between the three groups. Detailed transplantation characteristics are provided in Table 2.

### 3.2. Engraftment

No primary graft failure was observed in the three groups. One patient in both the URD-HSCT and Haplo-HSCT groups died of infection in aplasia (day 6 and 9 after transplantation). Using BM as the predominant graft source in the Haplo-HSCT group, median time to neutrophil engraftment was 18 days (range 13–34) in the Haplo-HSCT group compared to 14 days (range: 10–22) in the MRD-HSCT group (*p =* 0.002) and 14 days (range: 9–41) in the URD-HSCT group (*p =* 0.24; Supplemental Table S1). In addition, platelet recovery was significantly delayed in the Haplo-HSCT group, with 27 days (range 14–71) reported for this group compared to 11 days and 16 days in the MRD-HSCT and URD-HSCT groups (*p <* 0.001/*p <* 0.001, Supplemental Table S1).

### 3.3. Treatment-Related Toxicities and Infections

Severe non-hematological related toxicities (grade III–IV) in the Haplo-HSCT group (50%) were comparable to those of the MRD-HSCT (44%) and URD-HSCT groups (44%, *p =* 0.76), with mucositis (25%), creatinine elevation (19%) and transient elevation of liver enzymes (19%) as the most common ones in the Haplo-HSCT group (Table 3). Hemorrhagic cystitis grade I–II was more often detected following Haplo-HSCT (38%) compared to MRD-HSCT (6%, *p =* 0.03) and URD-HSCT (13%, *p =* 0.1; Table 3). In total, 3/8 (38%) patients at risk showed CMV reactivation in the Haplo-HSCT group compared to 2/11 (18%, *p =* 0.35) in the MRD-HSCT group and 0/8 (0%, *p =* 0.06) in the URD-HSCT group (Table 2). EBV reactivation was diagnosed in one patient in both the Haplo-HSCT and URD-HSCT groups (*p >* 0.99) and in four patients of the MRD-HSCT group (*p =* 0.14). No CMV-associated pneumonia or diarrhea and no post-transplantation lymphoproliferative disorder (PTLD) were diagnosed in the entire cohort. JC and/or BK infections occurred in five patients (31%) in the Haplo-HSCT group, in five patients (31%) in the URD-HSCT group (*p >* 0.99) and in two patients (13%) in the MRD-HSCT group (*p =* 0.17). Furthermore, 10 patients were diagnosed with invasive fungal infections, including two (13%) proven and four (25%) possible cases in the Haplo-HSCT group, one (6%) proven case in the MRD-HSCT group (*p =* 0.07) and one (6%) proven and two (13%) possible cases in URD-HSCT group (*p =* 0.50).

### 3.4. GvHD, Relapse and NRM

**Figure 1:**
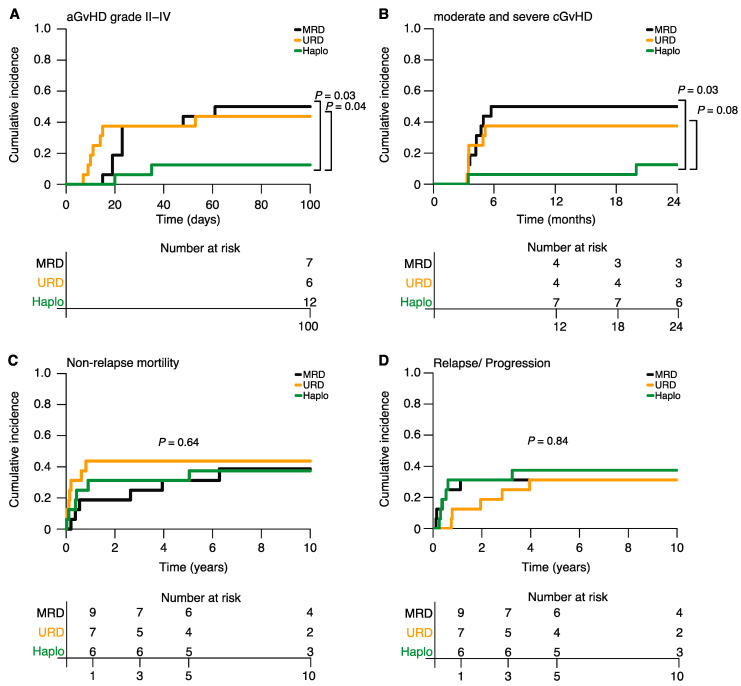
Cumulative incidences of acute GvHD (**A**), chronic GvHD (**B**), NRM (**C**) and disease relapse/progression (**D**) by donor type.

CI of acute GvHD grade II–IV at day 100 was significantly lower in the Haplo-HSCT group (13%) compared to the MRD-HSCT (50%, *p =* 0.03) and URD-HSCT groups (44%, *p =* 0.04; Figure 1A, Supplemental Table S2). CI of moderate and severe chronic GvHD at 12 and 24 months after Haplo-HSCT was 6% and 13%, respectively, and thus also remarkably lower than the corresponding incidences in the MRD-HSCT group (50%/50%, *p =* 0.01/0.03) and the URD-HSCT group (38%/38%, *p =* 0.04/0.08; Figure 1B, Supplemental Table S2). Due to contraindication or logistical issues, only three patients in the Haplo-HSCT and URD-HSCT groups and one patient in the MRD-HSCT group received pDLI. No difference in the intermediate-term 5-year and long-term 10-year NRM CI (Haplo: 31%/38%, MRD: 31%/38%, URD: 44%/44%, *p =* 0.69/0.64; Figure 1C, Supplemental Table S2) and disease relapse/progression CI (Haplo: 38%/38%, MRD: 31%/31%, URD: 31%/31%, *p =* 0.84/0.84; Figure 1D, Supplemental Table S2) was observed between the three groups. In total, 56% of patients in each group died within the first 5 years after allo-HSCT, with disease relapse/progression being the most frequent cause of death in the Haplo-HSCT (45%) and MRD-HSCT (45%) groups. In contrast, GvHD—the most common cause of death in the URD-HSCT group (56%)—was considered as the main cause of death in only one patient in the Haplo-HSCT group (Supplemental Table S3).

### 3.5. Outcome

**Figure 2:**
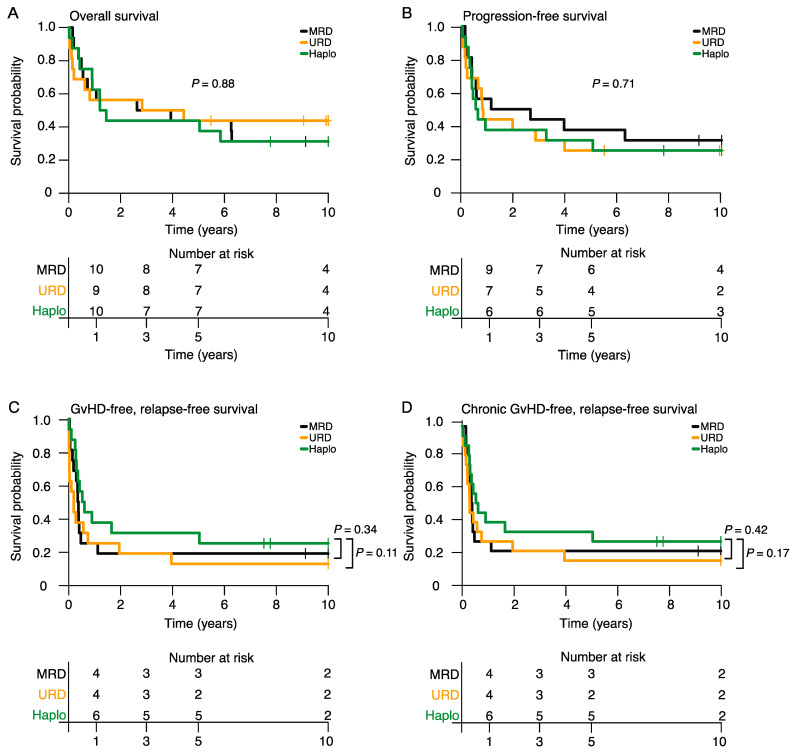
Kaplan–Meier estimates of overall survival (**A**), progression-free survival (**B**), GvHD-free and relapse-free survival (**C**) and chronic GvHD-free and relapse-free survival (**D**) by donor type.

With a median follow-up of >10 years for the entire cohort, intermediate-term 5-year OS was 44% (95% CI: 25–76) for the Haplo-HSCT group compared to 44% (95% CI: 25–76, *p =* 0.83) for the MRD-HSCT group and 44% (95% CI: 25–76, *p =* 0.89) for the URD-HSCT group (Figure 2A). Long-term OS was also comparable among the three groups (10-year OS: Haplo, 31% (95% CI: 15–65); MRD, 31% (95% CI: 15–65, *p =* 0.92); URD, 44% (95% CI: 25–76); *p* = 0.87). Similarly, there was no significant difference in the 5- and 10-year PFS among the three groups (Haplo: 31%/25%; MRD: 38%/31%, *p =* 0.75/0.71; URD: 25%/25%, p = 0.86/0.85; Figure 2B, Supplemental Table S4). Interestingly, patients undergoing Haplo-HSCT had a trend towards higher 5- and 10-year GRFS (31%/25%) and CRFS (31%/25%) compared to the URD-HSCT group (13%/13%, *p =* 0.09/0.11; 13%/13%; *p =* 0.14/0.17;Figure 2C,D, Supplemental Table S4).

## 4. Discussion

By performing a matched-pair analysis using age, disease status, lymphoma classification and performance status as matching criteria, our study provides a comprehensive and detailed assessment of Haplo-HSCT in patients with B and T cell lymphoma. Our results demonstrate that Haplo-HSCT is a curative option for patients with high-risk NHL that offers long-term remission and manageable toxicity rates. Having comparable outcomes among Haplo-HSCT, MRD-HSCT and URD-HSCT groups, lower GvHD incidences and rapid donor availability in the Haplo-HSCT group, our results further indicate that Haplo-HSCT is an effective alternative to URD-HSCT in patients with r/r NHL, particularly in patients presenting with non-remission disease.

In line with previous results, 2- and 3-year OS and PFS in the Haplo-HSCT group were comparable to those of the MRD-HSCT and URD-HSCT groups [19,21,22,33,34,35]. With a median follow-up >10 years, our study demonstrates for the first time that Haplo-HSCT is not inferior to HLA-matched HSCT in long-term disease control and survival. Considering the high-risk patient cohort including 88% with active disease at the time of HSCT initiation, it is noteworthy that around one-third of the patients who underwent Haplo-HSCT successfully achieved sustained disease control.

A critical issue faced in lymphoma Haplo-HSCT settings is disease relapse and progression. One of the major risk factors is the patient’s pre-transplantation disease status [12,36]. Patients with B and T cell NHL being chemo-resistant or achieving no CR prior to HSCT initiation frequently relapse before the durable graft-versus-leukemia (GvL) effect can be established [12,36,37]. The impact of specific conditioning regimens on outcome has been a matter of ongoing discussion. The NMC regimen has demonstrated feasibility and tolerance; however, it has been plagued by a significant drawback in the form of high relapse rates [17]. The intensification of conditioning by Luznik et al. was planned to overcome the lymphoma relapse in the original study [37], but relapse rates are still concerning and relapse remains a major cause of death [21,23,38]. Comparing relapse rates among the three groups in our high-risk cohort, we did not observe higher relapse rates in the Haplo-HSCT group (5 y CI of relapse/progress: Haplo, 38%; MRD, 31%; URD, 31%). Overall, relapse rates were similar to those reported by Kanate et al., including only ~40% patients with non-remission disease [19]. However, progression of disease was still the main cause of death within the first 5 years after allo-HSCT in the MRD-HSCT and Haplo-HSCT groups. Hence, there is room for improvement in conditioning regimens that control disease burden while minimizing treatment-related toxicities in patients with r/r disease.

Our study supports the finding of reduced incidences of GvHD in the Haplo setting [19,23]. We detected lower acute grade II–IV and moderate and severe chronic GvHD in the Haplo-HSCT group compared to the HLA-matched groups. This finding was independent of whether the HLA-matched recipients received ATG (URD-HSCT group) or not (MRD-HSCT group). The lower GvHD incidences might be explained by the use of PTCY as GvHD prophylaxis [19,23,25,26]. The utilization of PTCY-based GvHD prophylaxis is rapidly increasing due to its promising results in Haplo-HSCT. An analysis of the Center for International Blood and Marrow Transplant Research (CIBMTR) data comparing URD-HSCT and Haplo-HSCT, both using PTCY GvHD prophylaxis, in patients with acute leukemia or myelodyplastic syndrome demonstrated significantly higher risks of acute GvHD, NRM and inferior OS for RIC Haplo-HSCT but no survival difference in those receiving MAC regimens [39]. Focusing on lymphoma, Mussetti et al. recently demonstrated that URD-HSCT using PTCY had lower acute grade II–IV and chronic GvHD incidences compared to Haplo-HSCT [40]. However, the heterogeneity of the study population regarding age, lymphoma subtypes, GvHD prophylaxis, graft source, conditioning regimen and donor age should be considered. Additionally, no information regarding ABO compatibility was reported, and we do not know how the different centers performed donor selection. Prospective trials to identify the best GvHD prophylaxis in the HLA-matched setting (e.g., PROGRESS 3 trial) will shed light on this compelling question. Nevertheless, other donor-specific variables may also contribute to lower GvHD rates in our Haplo-HSCT group. While we did not observe any significant differences in ABO compatibility between the three groups, BM grafts, proven to decrease the risk of chronic GvHD [41], were more frequently used in the Haplo-HSCT group than in the HLA-matched groups. Additionally, we observed a significantly higher donor age in the MRD-HSCT group compared to the Haplo-HSCT and URD-HSCT groups. Importantly, lower GvHD incidences in the Haplo-HSCT group did not lead to increased relapse/progression rates in our high-risk patient collective, suggesting that the GvL effect after Haplo-HSCT is independent of chronic GVHD. Given the accumulating evidence of comparable OS and PFS between HLA-matched HSCTs and Haplo-HSCT in patients with NHL, compositive endpoints representing a more comprehensive picture of patient outcome, like GRFS and CRFS, should be considered when evaluating the impact of donor types [12,23].

While most studies comparing Haplo to HLA-matched donors in lymphoma primarily focused on outcomes, we additionally provide comprehensive treatment-related toxicity and infection analyses. Collectively, severe treatment-related toxicities did not significantly differ among the three groups. Hemorrhagic cystitis tended to be higher in the Haplo-HSCT group, probably due to the use of PTCY. While we did not observe significant differences in EBV, JC and BK infections among the three groups, less CMV infections were detected in the patients at risk in the MRD-HSCT group compared to the Haplo-HSCT group. Additionally, BM as graft source, resulting in delayed time to neutrophil engraftment, probably led to more invasive fungal infections in Haplo-HSCT group compared to the MRD-HSCT group. No difference in viral and fungal infections was observed between Haplo-HSCT and URD-HSCT. Overall, NRM rates were comparable among the three groups.

Novel immunotherapeutic treatment strategies including CAR T cells and bispecific antibodies have questioned the role of allo-HSCT in r/r lymphoma. However, despite the high overall response rates, long-term follow-up data indicate that CD19-targeted CAR T cells are likely to be curative for only a subset of patients with B cell lymphomas [2,42,43,44]. Moreover, given the short median follow-up for many CAR T cell and bispecific studies, time-consuming manufacturing procedures and the need for treatment alternatives after relapse, allo-HSCT remains a powerful curative option for patients with r/r B cell lymphomas. In particular, for patients with active disease and high tumor burden, rapid available and effective allogeneic regimens are one of the best and only options. Thus, although in the current study none of the patients had been pretreated with immunotherapeutic approaches, allo-HSCT remains a standard approach after the use of CAR T cells in current national and international guidelines [45,46]. Unlike B cell NHLs, CAR T cell therapies for T cell NHLs are in the early development phases, and allo-HSCT thus remains the standard-of-care option for r/r T cell NHL. In the future, clinical studies have the important and exciting task of combining and timing new therapeutic options with allogeneic regimens.

We are fully aware of the shortcomings of our study, including the small cohort size and the retrospective nature of our study. In addition, despite high matching quality, involving age, disease status, lymphoma classification and performance status, as well as mostly well-balanced patient and transplantation characteristics, some imbalances remain among the three groups. Furthermore, comparing intermediate-term and long-term outcomes necessitates extended follow-up time intervals, resulting in variations in the median year of transplantation in our study. However, in contrast to register-based analyses, our study provides an accurate and detailed comparison among the different transplantation settings, including GvHD and infection, and treatment-related toxicity analyses that are not impacted by institutional expertise and/or different local standards.

## 5. Conclusions

Collectively, given (i) comparable OS, PFS and NRM among the three groups, (ii) a trend towards higher GRFS and CRFS in the Haplo-HSCT group, (iii) remarkably lower acute and chronic GvHD incidences in the Haplo-HSCT group and, in particular, (iv) rapid donor availability, our data suggest that Haplo-HSCT is not only an effective treatment in patients with high-risk r/r NHL, but also should be favored over URD-HSCT in patients presenting with active disease requiring urgent treatment. To further elucidate optimal donor strategies including Haplo-HSCT as a first-choice decision, future prospective and controlled randomized studies are needed.

## Figures and Tables

**Table 1:** Patient characteristics.

	MRD (1)	URD (2)	Haplo (3)		*p* Value	
				1 vs. 3	2 vs. 3	Overall
Patients	16 (100)	16 (100)	16 (100)			
Patient age *, median (range), year	53 (42–62)	51 (44–67)	52 (45–66)	0.98	0.57	0.76
Patient sex						
Female	4 (25)	8 (50)	5 (31)	>0.99	0.47	0.31
KPS ≥ 80 *	16 (100)	16 (100)	16 (100)	>0.99	>0.99	>0.99
ECOG 0-I *	16 (100)	16 (100)	16 (100)	>0.99	>0.99	>0.99
Lymphoma classification *				>0.99	>0.99	>0.99
Indolent	2 (12.5)	2 (12.5)	2 (12.5)			
Aggressive	14 (87.5)	14 (87.5)	14 (87.5)			
Histology				0.79	0.88	0.83
Indolent B-NHL	2 (12.5)	2 (12.5)	2 (12.5)			
Aggressive T-NHL	4 (25)	2 (12.5)	2 (12.5)			
MCL	6 (37.5)	4 (25)	6 (37.5)			
DLBCL	4 (25)	8 (50)	6 (37.5)			
Status prior HSCT initiation *				>0.99	>0.99	>0.99
CR	2 (12.5)	2 (12.5)	2 (12.5)			
Active	14 (87.5)	14 (87.5)	14 (87.5)			
Prior therapy regimens				0.34	0.71	0.44
Median (range)	4 (3–7)	4 (2–7)	5 (2–7)			
Prior auto-HSCT	11 (69)	10 (62.5)	9 (56)			
Modified DRI score				0.85	0.55	0.66
Low	3 (19)	2 (12.5)	2 (13)			
Intermediate	4 (25)	7 (44)	4 (25)			
High	6 (37)	2 (12.5)	5 (31)			
Very high	3 (19)	5 (31)	5 (31)			
HCT score						
≥3	10 (62.5)	7 (44)	8 (50)	0.48	0.72	0.56
Median follow-up, year	10.4	10.2	10.5			

* served as matching criteria. Values in parentheses represent percentages if not indicated otherwise. CR, complete remission; DLBCL, diffuse large B cell lymphoma; DRI, Disease Risk Index; Haplo, haploidentical donor; HSCT, hematopoietic stem cell transplantation; KPS, Karnofsky performance score; MCL, mantle cell lymphoma; MRD, matched related donor; NHL, non-Hodgkin lymphoma; URD, matched unrelated donor.

**Table 2:** Donor and treatment characteristics.

	MRD (1)	URD (2)	Haplo (3)	*p* Value		
				1 vs. 3	2 vs. 3	Overall
Patients	16 (100)	16 (100)	16 (100)			
Donor age, median (range), year	48 (23–63)	28 (20–58)	27 (19–68)	0.009	0.86	0.002
Donor sex						
Female	6 (37.5)	4 (25)	12 (75)	0.03	0.005	0.12
D-R sex match						
Female—Male	5 (31)	2 (13)	8 (50)	0.28	0.02	0.07
Donor kindship				n.a.	n.a.	n.a.
Sibling	16 (100)	n.a.	4 (25)			
Children	n.a.	n.a.	10 (63)			
Parents	n.a.	n.a.	1 (6)			
Cousins	n.a.	n.a.	1 (6)			
ABO incompatibility				0.92	0.52	0.85
Major	1(6)	1(6)	1 (6)			
Minor	5 (31)	7 (44)	4 (25)			
None	10 (63)	8 (50)	11 (69)			
Stem cell source				<0.001	<0.001	<0.001
Bone marrow	1 (6)	0	11 (69)			
Peripheral blood	15 (94)	16 (100)	5 (31)			
Average graft cell dose (range)						
NC × 10^8^/kg BW	2.0	0	3.2 (1.7–4.4)	n.a.	n.a.	n.a.
CD34^+^ × 10^6^/kg BW	8.7 (3.3–14.5)	9.1 (4.7–14)	6.8 (2.9–14.3)	0.34	0.2	0.39
CMV R-D sero-status				0.42	0.77	0.78
Negative/negative	5 (31)	8 (50)	8 (50)			
Negative/positive	2 (13)	1 (6)	0			
Positive/negative	4 (25)	3 (19)	4 (25)			
Positive/positive	5 (31)	4 (25)	4 (25)			
Conditioning regimen				0.04	0.001	<0.001
Flu/CY/TBI	5 (31)	7 (44)	0			
Flu/CY ± others	6 (38)	5 (31)	0			
Flu/Mel ± others	0	3 (19)	16 (100)			
TBI ± others	5 (31)	0	0			
Others	0	1 (6)	0			
GvHD prophylaxis				<0.001	<0.001	<0.001
PTCY	0	0	16 (100)			
CNI—MMF ± others	13 (81)	15 (94)	16 (100)			
CNI—MTX ± others	1 (6)	0	0			
Sirolimus—MMF	2 (13)	1 (6)	0			
Median year of transplantation	2006	2008	2012			

Values in parentheses represent percentages if not indicated otherwise. Abbreviations: ATG, anti-thymocyte globulin; BW, body weight; CMV, cytomegalovirus; CNI, calcineurin inhibitors; CY, cyclophosphamide; D, donor; Flu, fludarabine; Haplo, haploidentical donor; Mel, melphalan; MMF, mycophenolic acid; MRD, matched related donor; MTX, methotrexate; NC, neutrophil count; n.a., not applicable, PTCY, post-transplantation cyclophosphamide; R, recipient; TBI, total body irradiation; URD, matched unrelated donor.

**Table 3:** Treatment-related toxicities.

	MRD (1)		URD (2)		Haplo (3)		*p* Value		
Grade	I–II	III–IV	I–II	III–IV	I–II	III–IV	1 vs. 3	2 vs. 3	Overall
GI tract									
Mucositis	7 (44)	2 (13)	6 (38)	2 (13)	8 (50)	4 (25)	0.46	0.32	0.62
Nausea/vomiting	14 (88)	1 (6)	12 (75)	1 (6)	12 (75)	0	0.23	0.56	0.56
Diarrhea	12 (75)	4 (25)	11 (69)	3 (19)	12 (75)	2 (13)	0.26	0.89	0.61
Liver									
Hyperbilirubinemia	3 (19)	2 (13)	4 (25)	3 (19)	8 (50)	1 (6)	0.17	0.28	0.32
Transient elevation of transaminases	2 (13)	1 (6)	4 (25)	2 (13)	6 (38)	3 (19)	0.09	0.57	0.31
Ascites	1 (6)	0	1 (6)	0	2 (13)	0	0.54	0.54	0.76
VOD	0	0	0	0	0	0	>0.99	>0.99	>0.99
Lung									
Dyspnea	2 (13)	3 (19)	1 (6)	6 (38)	1 (6)	1 (6)	0.43	0.1	0.25
Urogenital tract									
Creatinine elevation	3 (19)	2 (13)	6 (38)	1 (6)	7 (44)	3 (19)	0.20	0.43	0.39
Hemorrhagic cystitis	1 (6)	0	2 (13)	0	6 (38)	0	0.03	0.1	0.06
Skin									
Hand–foot syndrome	1 (6)	0	0	0	0	0	0.31	>0.99	0.36
Rash	1 (6)	1 (6)	0	0	0	0	0.34	>0.99	0.38
TAM	0	0	2 (13)	0	1 (6)	0	0.38	>0.99	0.34
Cardiovascular system									
Arrhythmia	0	3 (19)	2 (13)	3 (19)	1 (6)	1 (6)	0.36	0.43	0.48
CNS									
Headache	6 (38)	0	5 (31)	0	0	0	0.01	0.02	0.03
Confusion	4 (25)	2 (13)	3 (19)	2 (13)	2 (13)	1 (6)	0.50	0.71	0.83

Values in parentheses represent percentages. Abbreviations: CNS, central nervous system GI, gastrointestinal; Haplo, haploidentical donor; MRD, matched related donor; TAM, transplantation associated microangiopathy; URD, matched unrelated donor. VOD, veno-occlusive disease.

## Data Availability

Data used in this work are available upon reasonable request from the corresponding author.

## Supplementary Materials

The following supporting information can be downloaded at: https://www.mdpi.com/article/10.3390/cancers15215246/s1, Table S1: Engraftment after allo-HSCT; Table S2. Cumulative incidence of acute and chronic GvHD, disease relapse/progression and NRM; Table S3. Causes of death within the first 5 years after allo-HSCT; Table S4. Outcome after allo-HSCT.
