# Supplementary material for: PTCY-Based Haploidentical Donor Transplantation versus HLA-Matched Related and Unrelated Donor Transplantations in Patients with Refractory or Relapsed Lymphoma—A Matched-Pair Analysis
Source: Cancers (Basel). 2023 Oct 31;15(21):5246. doi: 10.3390/cancers15215246 (PMC10650710; doi:10.3390/cancers15215246)
Supplement: Supplementary file 1 [file cancers-15-05246-s001.zip › cancers-2641005-supplementary.pdf]

Supplementary Material

Supplementary Table S1. Engraftment after allo-HSCT.

	MRD (1)	URD (2)	Haplo (3)	P value		
				1 vs 3	2 vs 3	Overall
ANC > 500/µl						
Median days (range)	14 (10-22)	14 (9-41)	18 (13-34)	0.002	0.24	0.01
PLT > 20,000/µl						
Median days (range)	11 (6-22)	16 (5-26)	27 (14-71)	<0.001	<0.001	<0.001

ANC, absolute neutrophil count; *Haplo,* haploidentical donor; *MRD*, matched related donor; *PLT*, platelets; *URD,* matched unrelated donor.

Supplementary Table S2. Cumulative incidence of acute and chronic GvHD, disease relapse/progression and NRM

	MRD (1)	URD (2)	Haplo (3)	P value/Odd ratio		
				1 vs 3	2 vs 3	Overall
**aGvHD (II-IV) (95% CI)**						
Day +100	50 (48-52)	44 (46-48)	13 (12-14)	0.03/0.15	0.04/0.17	0.06
**cGvHD (moderate, severe)**						
12 months	50 (48-52)	38 (36-40)	6 (4-8)	0.01/0.06	0.04/0.12	0.04
18 months	50 (48-52)	38 (36-40)	6 (4-8)	0.01/0.07	0.04/0.17	0.04
24 months	50 (48-52)	38 (36-40)	13 (11-15)	0.03/0.17	0.08/0.40	0.09
**NRM, %**						
1-year	19 (18-20)	44 (42-46)	31 (29-33)	0.93/1.50	0.43/0.64	0.66
3-year	25 (23-27)	44 (42-46)	31 (29-33)	0.90/1.25	0.43/0.82	0.67
5-year	31 (29-33)	44 (42-46)	31 (29-33)	0.87/0.86	0.43/0.71	0.69
10-year	38 (36-40)	44 (42-46)	38 (36-40)	0.89/1	0.41/0.86	0.64
**Relapse/PD, %**						
1-year	25 (24-26)	13 (12-14)	31 (30-32)	0.76/2.08	0.51/2.5	0.82
3-year	31 (29-33)	25 (24-26)	31 (30-32)	0.77/1.33	0.56/1.04	0.85
5-year	31 (29-33)	31 (29-33)	38 (36-40)	0.77/1.68	0.55/0.96	0.84
10-year	31 (29-33)	31 (29-33)	38 (36-40)	0.77/1.6	0.55/0.8	0.84

aGvHD, acute graft-versus-host disease; *cGvHD*, chronic graft-versus-host disease; *Haplo,* haploidentical donor; *MRD*, matched related donor; *NRM*, non-relapse mortality; *PD*, progression of disease; *URD,* matched unrelated donor.

Supplementary Table S3. Causes of death within the first 5 years after allo-HSCT

	MRD	URD	Haplo
**No of deaths (%)**	**9 (100)**	**9 (100)**	**9 (100)**
Primary disease	4 (45)	2 (22)	4 (45)
Infection	2 (22)	2 (22)	3 (33)
GvHD	3 (33)	5 (56)	1 (11)
Toxicity	0	0	1 (11)

GvHD, graft-versus-host disease.

Supplementary Table S4. Outcome after allo-HSCT.

	MRD (1)	URD (2)	Haplo (3)	P value		
				1 vs 3	2 vs 3	Overall
**OS (95% CI)**						
1-year	63 (43-91)	56 (37-87)	63 (43-91)	0.76/1	0.81/0.77	0.77
3-year	50 (31-82)	50 (31-82)	44 (25-76)	0.86/1.3	0.92/1.3	0.85
5-year	44 (25-76)	44 (25-76)	44 (25-76)	0.83/1	0.89/1	0.83
10-year	31 (15-65)	44 (25-76)	31 (15-65)	0.92/1	0.87/1.22	0.88
**PFS (95% CI)**						
1-year	56 (37-87)	44 (25-76)	38 (20-71)	0.72/2.14	0.91/1.30	0.71
3-year	44 (25-76)	31 (15-65)	38 (20-71)	0.77/1.3	0.85/0.76	0.77
5-year	38 (20-71)	25 (11-58)	31 (15-65)	0.75/1.32	0.86/0.73	0.74
10-year	31 (15-65)	25 (11-58)	25 (11-58)	0.71/1.45	0.85/0.67	0.71
**GRFS (95% CI)**						
1-year	25 (11-58)	25 (11-58)	38 (20-71)	0.36/0.56	0.08/0.56	0.39
3-year	19 (7-52)	19 (7-52)	31 (15-65)	0.34/0.51	0.09/0.51	0.37
5-year	19 (7-52)	13 (3-46)	31 (15-65)	0.34/0.51	0.09/0.31	0.37
10-year	19 (7-52)	13 (3-46)	25 (11-58)	0.34/0.92	0.11/0.86	0.38
**CRFS (95% CI)**						
1-year	25 (11-58)	25 (11-58)	38 (20-71)	0.43/0.56	0.14/0.56	0.43
3-year	19 (7-52)	19 (7-52)	31 (15-65)	0.41/0.51	0.15/0.51	0.41
5-year	19 (7-52)	13 (3-46)	31 (15-65)	0.40/0.51	0.14/0.31	0.40
10-year	19 (7-52)	13 (3-46)	25 (11-58)	0.42/0.92	0.17/0.86	0.42

CI, confidence interval; *CRFS,* chronic graft-versus-host disease, relapse-free survival; *GRFS*, graft-versus-host disease, relapse-free survival; *Haplo,* haploidentical donor; *MRD*, matched related donor; *OS*, overall survival; *PFS*, progression-free survival; *URD,* matched unrelated donor.
